# Mr. Walter Adams

**Published:** 1906-03-10

**Authors:** 


### OBITUARY.

### MR. WALTER ADAMS.

Probably no class of institutions count amongst their staff a larger number of old and faithful servants of whom the world hears little or nothing, who devote the whole of their lives to the service of the sick, than hospitals.

The late Mr. Walter Adams was for thirty-five years connected with the secretary's office of the "Dreadnought" Seamen's Hospital at Greenwich. He commenced his service under Mr. Kemball Cook in 1871 as clerk, and rose to the office of assistant secretary and accountant in 1882, which office he has held ever since to the great advantage of the hospital. No one who had any dealings with the secretariat of the Seamen's Hospital could fail to be impressed by Walter Adams's personality, or to appreciate his courteous readiness to be of assistance and help to everybody always. He was one of the kindest and most liberal of men, and the amount of good deeds and personal service which Walter Adams rendered to people in distress or want would probably, if known, astonish even his most intimate friends. He was so quiet and retiring in disposition that his sterling worth and merit were apt to pass unrecognised except by those who were brought into the closest association with his work. His death came most unexpectedly and suddenly, and it has caused quite a shock amongst the staff, for no man will be more sincerely or deservedly mourned than the late Walter Adams. He was working in the office as usual on Saturday morning, and was dead on Tuesday. His age was 56. Speaking from personal knowledge and long association, the writer can testify that no hospital ever had a more faithful servant nor exemplary officer than Walter Adams proved himself to be in the service of the "Dreadnought" Seamen's Hospital at Greenwich.

